# Handbook of Treatment

**Published:** 1888-01

**Authors:** 


**Handbook of Treatment—Arranged as an Alphabetical Index of Diseases, to Facilitate Reference, and containing nearly One Thousand Formulæ.** By WILLIAM AITKEN, M. D., Edinburgh, F. R. S., Professor of Pathology in the Army Medical School; Examiner in Medicine for the Military Medical Services of the Queen; Fellow of the Sanitary Institute of Great Britain; Corresponding Member of the Royal Imperial Society of Physicians of Vienna; and of the Society of Medicine and Natural History of Dresden, etc. Edited, with Notes and Additions, by A. D. Rockwell, A. M., M. D., Late Electro Therapeutist to the New York State Woman's Hospital. E. B. Treat, publisher, 771 Broadway, New York. Price, $2.75.

There is, perhaps, no more striking characteristic of the medical practitioner of to-day, and none better illustrating the pervading spirit of the age, than the universally-observed tendency among medical men to shun (in medical literature) the unrealities of theoretical discussion, and to appropriate with avidity only facts which they can instantly transform into working force. A

book which is at once concise and comprehensive, arranged so that the practitioner, given a disease to treat, may have before him in a nutshell the latest treatment recommended by the best authorities; and a book which is, above all else, *a book of* TREATMENT, is here offered to the profession. It is composed of the chapters on *Treatment* compiled from the seventh (latest) edition of Dr. Aitken's classical work on the "Science and Practice of Medicine," which chapters have been revised and rearranged by Dr. Aitken, so as to make them more available for reference. The work not only embraces the experience of its distinguished author, but also that of many widely-known authorities.

